# β-blockers and risk of all-cause mortality in patients with chronic heart failure and atrial fibrillation—a meta-analysis

**DOI:** 10.1186/s12872-019-1079-2

**Published:** 2019-06-03

**Authors:** Tianyu Xu, Yuli Huang, Haobin Zhou, Yujia Bai, Xingfu Huang, Yunzhao Hu, Dingli Xu, Yuhui Zhang, Jian Zhang

**Affiliations:** 1 State Key Laboratory of Cardiovascular Disease, Heart Failure Center, Fuwai Hospital, National Center for Cardiovascular Diseases, Chinese Academy of Medical Sciences and Peking Union Medical College, 167 Beilishi Road, Beijing, 100037 China; 2 Department of Cardiology, Shunde Hospital, Southern Medical University, F Penglai Road, Daliang Town, Shunde District, Foshan, 528300 China; 3 State Key Laboratory of Organ Failure Research, Department of Cardiology, Nanfang Hospital, Southern Medical University, 1838 North Guangzhou Avenue, Guangzhou, 510515 China

**Keywords:** Heart failure, Atrial fibrillation, Beta blockers, All-cause mortality

## Abstract

**Background:**

Effects of β-blockers on outcomes in patients with chronic heart failure (CHF) and atrial fibrillation (AF) is still in controversy.

**Methods:**

Searching was conducted by using keywords “atrial fibrillation”, and “heart failure” in PubMed, MEDLINE and Embase databases before November 30, 2017. Prospective studies [i.e. randomized control trials (RCTs), post-hoc analysis of RCTs, prospective cohort studies and registry studies] that studied the effect of β-blockers and all-cause mortality in patients with CHF and AF were included. The analysis was stratified by study design.

**Results:**

We identified 12 studies, including 6 post-hoc analysis of RCTs and 6 observational studies (including prospective registry studies and prospective cohort studies), which enrolled 38,133 patients with CHF and AF. Overall, β-blockers treatment was associated with significant decrease in all-cause mortality [Risk Ratio (RR) =0.73; 95% Confidence Interval (CI) 0.65–0.82, *P* < 0.001]. When stratified by study design, β-blockers treatment was associated with 34% reduction in patients with CHF and AF in observational study (RR = 0.66; 95% CI 0.58–0.76, *P* < 0. 001), but not in post-hoc analysis of RCT (RR = 0.87; 95% CI 0.74–1.02, *P* = 0.09).

**Conclusions:**

β-blockers treatment was associated with significantly decrease the risk of all-cause mortality in patients with AF-CHF and it was only seen in observational study group, but not in subgroup analysis of RCT group. Further large RCTs are required to verify the effect of β-blockers treatment on patients with CHF and AF. The main limitation of this study is the lack of individual data on patients in each study.

**Electronic supplementary material:**

The online version of this article (10.1186/s12872-019-1079-2) contains supplementary material, which is available to authorized users.

## Background

Chronic heart failure (CHF) remains a public health problem attaching growing attention since recent years, for its high prevalence (with an increase 46% from 2012 to 2030 in U.S.A.) and low 5-year survival rates (with estimating 50% in U.S.A.) [1, 2], which are much worse than some types of cancers [3]. Atrial fibrillation (AF) is one of the most common complications of CHF, with report of prevalence up to 50% in CHF patients [4, 5]. CHF and AF could coexist and interact with each other, promoting the development of cardiac dysfunction and increasing the risk of mortality [6, 7]. It has been reported that CHF with new-onset AF had greater mortality in 1 year [5].

β-blockers play an important role in treatment of heart failure with reduced ejection fraction (HFrEF). Large amounts of studies confirmed that it could decrease the rate of mortality and hospital readmission for HF of HFrEF in sinus rhythm (SR) [8–10] and β-blockers had been recommended as Class IA in guidelines in treating those CHF with SR patients by both U.S.A. and Europe [4, 11]. Also, β-blockers is the first-line rate control treatment in AF and has received a Class IB recommendation in treating patients with AF with left ventricular ejection fraction (LVEF) ≥ 40% or < 40% in the latest recommendation in Europe [12]. However, it still lacks direct and strong evidence from data of large randomized control trials (RCTs) which are originally designed for patients with CHF and AF and there has been a controversy about the benefit of β-blockers in treating CHF patients with AF. A meta-analysis with four RCTs indicated that β-blockers did not decrease the risk of all-cause mortality and HF hospitalization in CHF patients with AF [10]. Also, according to individual patient-level meta-analysis conducted by β-Blockers in Heart Failure Collaborative Group, which included data from RCTs, β-blockers were not associated with the with decreasing the risk of mortality, nor the hospital admission outcomes [9], regardless of LVEF [13] or heart rate (HR) [14]. However, registry study indicated an opposite view that β-blockers treatment could decrease all-cause mortality in patients with both CHF and AF. Recently, the European Society of Cardiology-Heart Failure (ESC-HF) Registry demonstrated that β-blockers could reduce the all-cause mortality in CHF and AF patients [Hazard ratio: 0.52; 95% Confidence Interval (CI) 0.31–0.89; *p* = 0.02], especially in the group with patients’ HR between 80 and 109 b.p.m. (beats per minute) [15]. Similarly, findings from one cohort study also support the idea that β-blockers could show a reduction of all-cause mortality in AF and CHF groups (Hazard ratio: 0.63, 95% CI: 0.50–0.79) [16].

Due to these inconsistent results in RCTs and registry studies, we performed a meta-analysis stratified by study design; to examine the effect of β-blockers on outcomes (i.e. all-cause mortality, cardiovascular mortality and hospitalization for HF) in CHF combined AF patients.

## Method

### Search strategy

Search was performed according to the recommendations of the Meta-Analysis of Observational Studies in Epidemiology Group [17]. We searched the PubMed, MEDLINE and Embase databases. We used the keywords “atrial fibrillation”, and “heart failure” or “cardiac dysfunction” or “heart dysfunction” or “cardiac failure” or “heart weakness”, and “beta blockers” or “adrenergic beta antagonists” or “bisoprolol” or “nebivolol” or “carvedilol” or “bucindolol” or “metoprolol” or “atenolol” or “metoprolol CR/XL” (Additional files 8 and 9). The search deadline was November 30, 2017. We further manually reviewed the reference lists of eligible studies. The search was restricted to human studies, but there were no language or publication form restrictions.

### Inclusion and exclusion criteria

The inclusion criteria of studies for analysis were: (1) prospective studies, including RCTs, post-hoc analysis of RCT, prospective cohort studies and registry studies; (2) patients diagnosed with AF (documented mainly by electrocardiography at baseline) and CHF (with combination of symptoms and cardiac dysfunction proven by echocardiogram); (3) inclusion of CHF combined AF patients aged≥18 years; (4) reported data of all-cause mortality or data of all-cause mortality and cardiovascular mortality or hospitalization for HF associated with β-blockers treatment.

Studies were excluded if: (1) patients who were not diagnosed with CHF and AF; (2) no controlled group (i.e.no comparison between β-blocker and other arrhythmia drugs or placebo); (3) no data were available for clinical outcomes or data were reported as composite endpoints, but not specified for all-cause mortality; (4) data were derived from the same study.

The primary outcome was all-cause mortality. The secondary outcomes were hospitalization for worsening HF and cardiovascular mortality.

### Data extraction

Two investigators (XT and HY) independently conducted literature searches, reviewed the potential articles, and abstracted data from eligible studies. Discrepancies were resolved by discussion with other investigators.

All available data were extracted from included studies, including crude outcome data and adjusted analyses, comprising multivariate adjustment and propensity matched data. If studies reported both unadjusted data and multivariate adjusted data, we only extracted multi-adjusted data.

### Quality assessment of studies

We assessed the risk of bias with the Cochrane Collaboration’s risk of bias tool for randomized control trials [18] and the risk of bias assessment tool for non-randomized studies [19], both addressing criteria about selection bias, blinding, measurement of exposure and outcome and reporting selectivity.

### Synthesis and analysis

The primary analysis was the relative risks or hazard ratios of all-cause mortality associated with β-blockers treatment. Crude or adjusted relative risks or hazard ratios and 95% CIs were logarithmically transformed. The corresponding standard errors (SEs) were calculated to stabilize the variance and normalize the distribution. If more rigorous analytic methodology, such as propensity score-matched analysis, was reported in the included studies, these data were used for analysis. We used inverse variance method to combine the calculated log risk ratios (RRs) and SEs. I^2^ statistics was used to test heterogeneity. Values of I^2^ > 50% were considered to be significantly heterogeneous. A random effects model was used if there was significant heterogeneity in the pooled estimation. Otherwise, a fixed effects model was used. Subgroup analyses of primary and second analysis were performed stratified by study design (Subgroup analysis of RCTs vs. observational studies). Sensitivity analyses were conducted by omitting 1 study at a time and recalculating the pooled RRs. Publication bias was assessed by inspecting funnel plots in which the natural log of RR was plotted against its SE, and further tested by Egger test and Begg test [20]. *P* values were 2-tailed, and statistical significance was set at 0.05. All analyses were conducted using RevMan [21] (Version 5.3; The Cochrane Collaboration, Copenhagen, Denmark) and Stata software (Version 12.0; Stata Corp LP, College Station, TX).

## Results

### Studies retrieved and characteristics

**Fig. 1:**
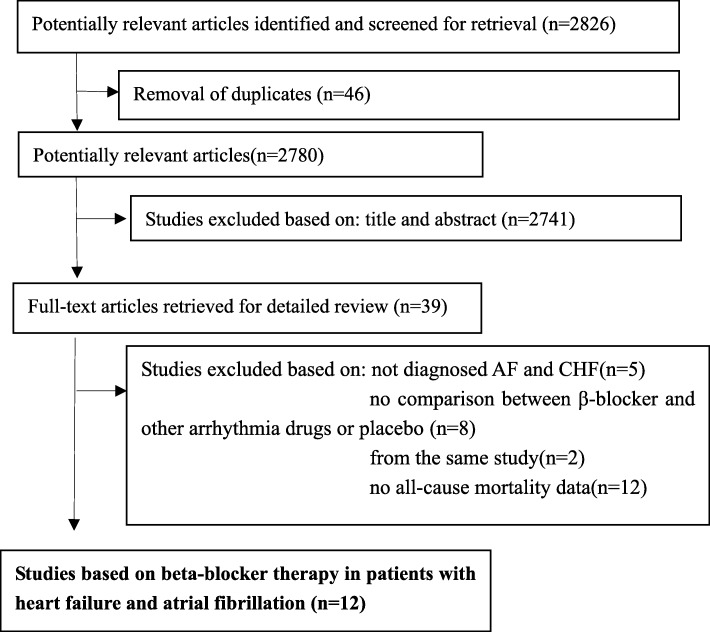
Flow of papers through review.AF: atrial fibrillation; CHF: chronic heart failure; CIs: confidence intervals; RRs: relative risk

The search retrieved 2, 826 manuscripts initially. After screening titles, abstracts and full text, we excluded 46 articles due to duplicates, 2, 741 articles on account of irrelevance, 5 articles because of patients in which were not diagnosed with AF and CHF, 8 articles for no controlled group, 12 articles for no all-cause mortality data, 2 articles for from the same study, and finally, 12 studies, including 38, 133 patients were included for analysis in this study [15, 16, 22–31] (Fig. 1). Table 1 summarizes the major differences in key characteristics. Six studies reported data from post-hoc analysis of RCTs [22–27] and six were observational studies [15, 16, 28–31], including 1 cohort study [16] and 5 registry studies [15, 28–31]. The sample size ranged from 136 to 23, 896 and the mean age of patients of included studies was from 62 to 77 years. Three studies [16, 22, 31] use propensity score matching to report the risk of mortality, three studies [15, 24, 30] analyzed data in single Cox-regression model and one [23] used log-rank test. In post-hoc analysis of RCT group, baseline HR ranged from 79 b.p.m. to 87 b.p.m., the mean reduction of HR after treatment was approximately 12 b.p.m., and HR at the end of follow-up time was around 75 b.p.m. [22, 23, 25–27]. However, in one observational study, Abi et al. indicated the baseline HR of AF and CHF patients was 115 b.p.m. [28], which was much higher than RCT group. In observational studies, baseline HR was higher than post-hoc analysis of RCTs. The follow-up duration ranged from 1 to 4.5 years (Additional files 7 and 10).

**Table 1:** Characteristics of included studies

Study	Country	Study Design	Mean follow-up, years	Sample (Women, %)	Mean age, years	LVEF,%	End points	Statistical Methods	Mean baseline HR, b.p.m.	Baseline BBs	Reduction HR at follow-up, b.p.m.	Follow-Up BBs
Fauchier 2009 [29]	France	Multicenter registry study	2.4	1269 (39%)	74	48 ± 17	all-cause mortality	Logistic regression and Multivariate Cox regression	NA	NA	NA	NA
Abi et al. 2018 [28] (Gulf Survey)	Qatar	Multicenter registry study	1	334 (34%)	62	< 40	all-cause mortality and hospitalization for HF	Multivariable logistic regression analyses	115	NA	NA	NA
Li 2015 [30] (Swedish Heart Failure Registry)	Sweden	Multicenter registry study	2.4	7392 (33.2%)	75.7	< 40	all-cause mortality	Multivariate Cox regression	NA	NA	NA	NA
Nielsen 2016 [31]	Demark	Multicenter registry study	3.1	23,896 (42.8%)	78	NA	all-cause mortality	Multivariate Cox regression with propensity score matching and Inverse Probability– Weighted	NA	NA	NA	NA
Ozieranski 2017 [15] (ESC-HF Registry)	Poland	Registry study	1.1	797 (27.5%)	72	< 40	all-cause mortality and hospitalization for HF	Cox regression	NA	NA	NA	NA
Yu 2017 [16]	Korea	Cohort study	4.5	1516 (50.6%)	69.2	NA	all-cause mortality	Multivariate Cox regression with propensity score matching	NA	NA	NA	NA
Cadrin- Tourigny 2016 [28] (AF-CHF)	Canada	post-hoc analysis of RCT	3.1	Cadrin- Tourigny 2016 (AF-CHF)	70	< 35	all-cause mortality, cardiovascular mortality and hospitalization for HF	Multivariate Cox regression with propensity score matching	79	NA	NA	NA
Joglar 2001 [23] (US Carvedilol)	U.S.A	post-hoc analysis of RCT	0.5	136 (10%)	65	< 35	all-cause mortality	log-rank test	87	6.25 mg of carvedilol twice daily	−13	25 mg of carvedilol twice daily
Kao 2013 [24] (BEST)	U.S.A	post-hoc analysis of RCT	2	303 (9%)	65.6	< 35	all-cause mortality	Cox regression	80	3 mg of bucindolol twice daily	−6.9	50 mg of bucindolol twice daily
Mulder 2012 [26] (SENIORS)	U.S.A	post-hoc analysis of RCT	1.8	738 (35.5%)	77	< 35	all-cause mortality and hospitalization for HF	Cox regression	84	1.25 mg of nebivolol daily	−11	10 mg of nebivolol daily
Lechat 2001 [25] (CIBIS II)	U.S.A	post-hoc analysis of RCT	1.3	521 (82.9%)	62.5	< 35	all-cause mortality, cardiovascular mortality and hospitalization for HF	Multivariate Cox regression	88	1.25 mg of bisoprolol daily	−8.8	10 mg of bisoprolol daily
Van Veldhuisen 2006 [27] (MERIT-HF)	U.S.A	post-hoc analysis of RCT	1	556 (13%)	65.7	< 40	all-cause mortality and hospitalization for HF	Multivariate Cox regression	84	12.5 mg of metoprolol CR/XL daily	−14.8	154 mg metoprolol CR/XL daily

### Association between β-blockers treatment and risk of all-cause mortality

**Fig. 2:**
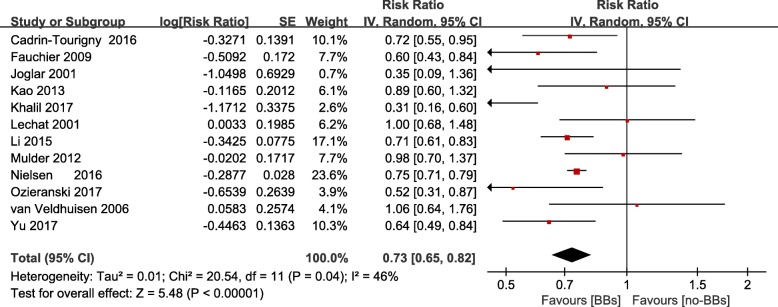
Frost plot of the comparison of β-blockers treatment versus no β-blockers treatment in patients with chronic heart failure and atrial fibrillation, outcomes: all-cause mortality. BBs: β-blockers; CI: confidence intervals; SE: standard error

Although the heterogeneity among the included studies was not significant (I^2^ = 46%), we still used random-effect models in analysis to provide conservative outcome. Overall, in HF combined AF patients, β-blockers treatment was associated with significant decrease in all-cause mortality (RR = 0.73; 95% CI 0.65–0.82, *P* < 0.001) (Fig. 2, Additional file 2). Sensitivity analysis showed that the result was not influenced by omitting 1 study at a time.

### All-cause mortality in β-blockers treatment modified by study design

**Fig. 3:**
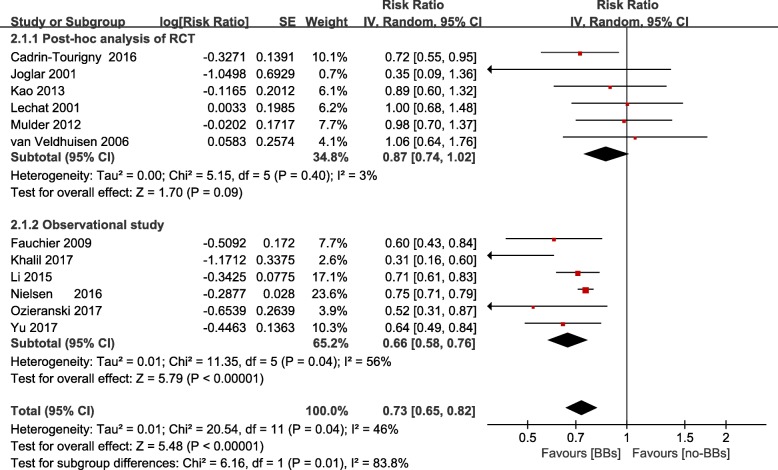
Frost plot of the comparison of β-blockers treatment versus no β-blockers treatment in patients with chronic heart failure and atrial fibrillation. Stratified on study design, outcomes: all-cause mortality. BBs: β-blockers; RCT: randomized control trial; CI: confidence intervals; SE: standard error

Pooled data from these studies showed that in observational study group, β-blockers treatment was significantly associated with 34% decrease of all-cause mortality (RR = 0.66; 95% CI 0.58–0.76, P < 0. 001), whereas the reduction was not seen in RCT group (RR = 0.87; 95% CI 0.74–1.02, *P* = 0.09) (Fig. 3, Additional file 5).

### Association between β-blockers treatment and risk of cardiovascular mortality

Two (both were post-hoc analysis of RCTs [22, 26]) studies reported data of β-blockers treatment in cardiovascular mortality, including 1, 393 patients. The analysis showed β-blockers treatment was not associated with a reduction of cardiovascular mortality (RR = 0.83; 95% CI 0.65–1.06, *P* = 0.14) (Additional files 1 and 3).

### Association between β-blockers treatment and risk of hospitalization for HF

**Fig. 4:**
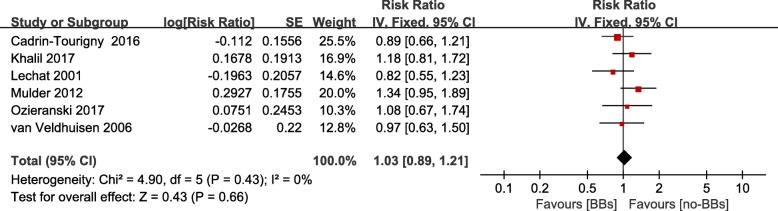
Funnel plot of the comparison of β-blockers treatment versus no β-blockers treatment in patients with chronic heart failure and atrial fibrillation, outcomes: heart failure hospitalization. BBs: β-blockers; CI: confidence intervals; SE: standard error

**Fig. 5:**
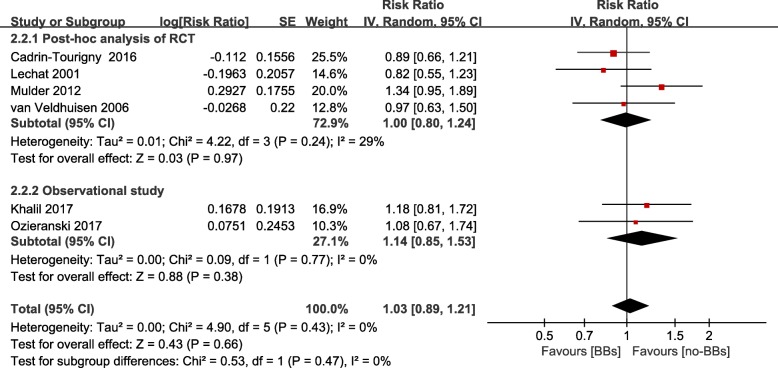
Frost plot of the comparison of β-blockers treatment versus no β-blockers treatment in patients with chronic heart failure and atrial fibrillation. Stratified on study design, outcomes: heart failure hospitalization. BBs: β-blockers; RCT: randomized control trial; CI: confidence intervals; SE: standard error

Six (four were post-hoc analysis of RCTs [22, 25–27] and two were observational studies [15, 28]) studies showed the effect of β-blockers treatment on hospitalization for HF. Including 3, 601 patients, pooled data indicated that β-blockers treatment was not associated with a reduction of hospitalization for HF (RR = 1.03; 95% CI 0.89–1.21, *P* = 0.66) (Fig. 4, Additional file 4). And the result was also seen when modified by study design (RCTs: RR = 1.00; 95% CI 0.83–1.19, *P* = 0.24; observational studies: RR = 1.14; 95% CI 0.85–1.53, *P* = 0.77) (Fig. 5, Additional file 6).

## Discussion

The main finding of the present meta-analysis indicates that β-blockers treatment could decrease the risk of all-cause mortality in patients with CHF and AF, but did not reduce the risk of cardiovascular mortality and HF hospitalization. We also found that β-blockers treatment only shows a reduction in observational study group, whereas the reduction in subgroup analysis of RCT group was not significant.

The effect of β-blockers treatment in patients with CHF and AF has been reported by several meta-analyses. One meta-analysis with four RCTs indicated that β-blockers did not decrease the risk of all-cause mortality and HF hospitalization in CHF patients with AF [10]. Another previous individual-patient-data meta-analysis performed by Kotecha et al., which included ten RCTs with 18,254 CHF patients (containing 3066 with AF), also showed that β-blockers treatment had no effect in reducing all-cause mortality in patients who had both AF and CHF [9]. It should be noted that RCTs included in these meta-analyses was originally designed for patients with CHF and patients with CHF and AF only comprised around 19% of the whole group. So there may be a less power to detect benefits of β-blockers treatment in patients with CHF and AF. In order to have a more comprehensive understanding, we not only included data from but also from observational studies in this meta-analysis. Besides, in our study, we used a wider search strategy with more search terms, including “atrial fibrillation”, “heart failure”, “cardiac dysfunction”, “heart dysfunction”, “cardiac failure”, “heart weakness”, “beta blockers”, “adrenergic beta antagonists”, “bisoprolol”, “nebivolol”, “carvedilol”, “bucindolol”, “metoprolol”, “atenolol” and “ metoprolol CR/XL” whereas only “atrial fibrillation”, “heart failure”, “beta blockade”, “beta-blocker therapy” and “medical therapy” were used to detect associated studies in the prior meta-analysis. We believe that our wider search strategy is important for meta-analysis to avoid missing potentially relevant studies.

Meta-analyses may be biased when the literature search fails to identify all relevant studies.

Several explanations may be contributed the difference in these studies. First, the dose of beta blockers may be different in subgroup analysis of RCTs and observational studies. Patients who receive higher doses of β-blockers may have more chance exposed to its side effects such as hypotension and bradyarrhythmias [32], which may counterbalance its benefits in improving conditions in AF and CHF patients. As shown in post-hoc analysis of RCTs, β-blockers were required to titrated to target dose or the tolerance dose for patients in follow-up period which were 25 mg twice daily [23], 10 mg of nebivolol daily [26], 10 mg of bisoprolol daily [25], 154 mg metoprolol CR/XL daily [27], respectively. The individual-patient-data meta-analysis by Kotecha et al. showed that 72.1% patients with AF and CHF received maximum does of β-blockers [9]. However, in observational studies, which were much more revealing the medication prescription for patients in real world, the prescribed dosage of β-blockers may be depended on conditions of each patient and be more individualized, so dosages of β-blockers for patients may not be as high as those in RCTs. In one registry, Li et, al pointed out that only 30% of patients in AF and CHF treating with β-blockers reached its target does [30]. The target does of β-blockers in treating patients with CHF may not benefit patients with CHF and AF. A previous study showed that 50% of the target does of β-blockers linked with a better prognosis in patients in AF and HFrEF [33]. Up to now, we still lack studies to learn which dose of β-blockers could give a better prognosis to patients with CHF and AF. Second, the baseline HR and achieved HR with β-blockers treatment may be different in post-hoc analysis of RCTs and observational studies. In RACE (Rate Control Efficacy in Permanent Atrial Fibrillation) II study, results showed that permanent AF patients in strict rate control group (resting HR ≤ 80 b.p.m., and a HR ≤110 b.p.m. during moderate exercise) was not better than those in lenient rate control (resting HR < 110 b.p.m.) in all-cause mortality [34]. An observational study pointed out that in patients with CHF and AF, each increase in resting HR of 10 b.p.m. at baseline was associated with a 7% decrease in mortality per year and HR < 73b.p.m. in patients with CHF and AF seemed to have a worse survival [35]. Likewise, a registry study showed that AF and CHF patients with HR control between 80b.p.m.-109b.p.m. had better prognosis compared with other two groups (< 80 b.p.m. and ≥ 110b.p.m.) [15]. Thus, different benefits reported in subgroup analysis of RCTs and observational studies may be due to the relatively low controlled heart rate in post-hoc analysis of RCTs, which would lead to side effects of β-blockers such as atrioventricular block. It is suggested that patients with HF and AF should pay attention to the ventricular rate, and the optimal dosage of β-blockers should be the dose that helps patients achieve the target ventricular rate, but not the dose designed in RCT which neglect patients’ target ventricular rate. Third, we also found that except for β-blockers, other medicines treating for AF and CHF may be different in both groups. For example, patients in post-hoc analysis of RCT group seemed to more likely to receive digoxin (mean 65.4%) than those in registry study group (mean 29.5%). It has been demonstrated that digoxin could increase the risk of all-cause mortality in AF patients with or without CHF [36]. As digoxin has negative effect on atrioventricular conduction [37], the therapy combining digoxin and β-blockers may exacerbate the possibility of atrioventricular block. Thus, in treating patients with AF and HF, clinicians should be more cautious when using combined medications of digoxin and β-blockers and avoid excessive dosage of these two drugs.

According to our analysis, β-blockers could reduce the risk of all-cause mortality among AF-CHF patients and should be considered as the first-line therapy for controlling rate. However, we could not ignore that there was not a significantly reduction in cardiovascular mortality and HF hospitalization when using β-blockers treatment, which require us to further investigate an optimal treatment scheme for patients with AF and CHF [38–40]. As previous studies mentioned, rate control were equivalent to rhythm control in reducing rates of all-cause mortality, cardiovascular mortality, thromboembolism in patients with AF, as well as in patients with AF and HFrEF [41]. Also, a study indicated that side-effect of drugs in both rate-control therapy and rhythm-therapy may contribute to progression of diseases and worse prognosis in AF-CHF patients [42]. However, recent report from Catheter ablation versus standard conventional treatment in patients with left ventricular dysfunction and atrial fibrillation (CASTLE-AF) trial demonstrated that AF ablation could significantly reduce composite endpoints of all-cause mortality and hospitalization for worsening HF in 60 months of follow-up (hazard ratio 0.62; 95% CI 0.43–0.87 *P* = 0.006) in enrolled AF and CHF patients [43]. Besides, previous studies showed that AF ablation was superior to rate control using medications in improving left ventricular function [44]. So AF ablation may become first choice for treating patients with AF and CHF in future, but its benefits need further studies.

We should note that our meta-analysis include both subgroup analysis of RCTs and observational studies and the outcome in two groups were different when analysis in stratifying by study design. Although large RCTs are considered as the highest level of evidence in professional societies, potential bias, such as patient selection and enrollment cannot be completely avoided. Furthermore, clinical heterogeneity (e.g different baseline characteristics, risk profile of patients and different pharmacological profiles) between studies included in individual patient data meta-analysis may also made the combined results misleading. Despite their often stated limitations, large sample observational studies can provide valuable information, which are critical to posing relevant questions in real world practice and help to inform the planning and design of RCTs. Taken these evidence together, we considered that: (1) Beta-blockers are still the first line medications for heart rate control in patients with HF and AF; (2) The effect on mortality of beta-blockers in patients with HF and AF should be furthermore evaluated based on HR strata.

### Limitations

There are several limitations of our meta-analysis which should be considered. First, as there was not RCTs were not initially designed for patients with AF and CHF, we could only include post-hoc analysis of RCTs which was originally designed for CHF patients and that may limit our understanding of the real effect of β-blockers on patients with CHF and AF. Second, the LVEF of patients in most articles we included was < 40% and we lacked sufficient data on patients whose LVEF ≥40%, so it is hard for us to extrapolate the conclusion to the whole population of AF and CHF which contain both heart failure with perserved ejection fraction and HFrEF. Third, since there was not sufficient data or individual data provided in the included studies, we were not able to perform more subgroup analysis except for study design, and some confounding factors maybe underestimated. Forth, included articles did not report the whether patients were still on treatment during follow-up period, so it is hard for us to evaluate if the compliance would affect the results and how it could affect the result. Fifth, although we adopt random model analysis, we were still unable to avoid the inherent heterogeneity from different articles due to their different follow-up durations, treatments and population, etc. Sixth, thought we did an entire search in published data, we could not include those unpublished articles and it is needed for us to give further analysis.

## Conclusion

β-blockers treatment was associated with significantly decrease the risk of all-cause mortality in patients with AF and CHF but not in reducing rates of cardiovascular mortality and HF hospitalization. The association between β-blockers treatment and the reduction of all-cause mortality was only seen in observational study group, whereas it was not significant in RCT group. Further RCTs targeting AF and CHF patients with β-blockers therapy as well as studies of new therapy, such as AF ablation, are needed for our better understanding of the management of patients with AF and CHF.

## Additional files

Additional file 1: Frost plot of the comparison of β-blockers treatment versus no β-blockers treatment in patients with chronic heart failure and atrial fibrillation, outcomes: cardiovascular mortality. BBs: β-blockers; CI: confidence intervals; SE: standard error. (PDF 212 kb)

Additional file 2: Funnel plot of the comparison of β-blockers treatment versus no β-blockers treatment in patients with chronic heart failure and atrial fibrillation, outcomes: all-cause mortality. BBs: β-blockers; CI: confidence intervals; SE: standard error. (PDF 29 kb)

Additional file 3: Funnel plot of the comparison of β-blockers treatment versus no β-blockers treatment in patients with chronic heart failure and atrial fibrillation, outcomes: cardiovascular mortality. BBs: β-blockers; CI: confidence intervals; SE: standard error. (PDF 28 kb)

Additional file 4: Funnel plot of the comparison of β-blockers treatment versus no β-blockers treatment in patients with chronic heart failure and atrial fibrillation, outcomes: all-cause mortality. BBs: β-blockers; CI: confidence intervals; SE: standard error. (PDF 30 kb)

Additional file 5: Funnel plot the comparison of β-blockers treatment versus no β-blockers treatment in patients with chronic heart failure and atrial fibrillation. Stratified on study design, outcomes: all-cause mortality. BBs: β-blockers; CI: confidence intervals; SE: standard error. (PDF 67 kb)

Additional file 6: Funnel plot of the comparison of β-blockers treatment versus no β-blockers treatment in patients with chronic heart failure and atrial fibrillation. Stratified on study design, outcomes: heart failure hospitalization. BBs: β-blockers; RCT: randomized control trial; CI: confidence intervals; SE: standard error. (PDF 67 kb)

Additional file 7: Risk of bias graph. (PDF 238 kb)

Additional file 8: Search strategy for pubmed. (DOCX 14 kb)

Additional file 9: Search strategy for embase. (DOCX 13 kb)

Additional file 10: Tests for Publication Bias. (DOCX 16 kb)

## Abbreviations

AF: Atrial fibrillation
BB: β-Blockers
CASTLE-AF: Catheter ablation versus standard conventional treatment in patients with left ventricular dysfunction and atrial fibrillation
CHF: Chronic heart failure
CI: Confidence interval
ESC-HF: European society of cardiology-heart failure
HF: Heart failure
HFrEF: Heart failure with reduced ejection fraction
HR: Heart rate
LVEF: Left ventricular ejection fraction
NA: Not available.
RACE: Rate control efficacy in permanent atrial fibrillation
RCTs: Randomized control trials
RRs: Risk ratios
SEs: Standard errors
