# Supplementary figures and images for: β-blockers and risk of all-cause mortality in patients with chronic heart failure and atrial fibrillation—a meta-analysis
Source: BMC Cardiovasc Disord. 2019 Jun 3;19:135. doi: 10.1186/s12872-019-1079-2 (PMC6547467; doi:10.1186/s12872-019-1079-2)

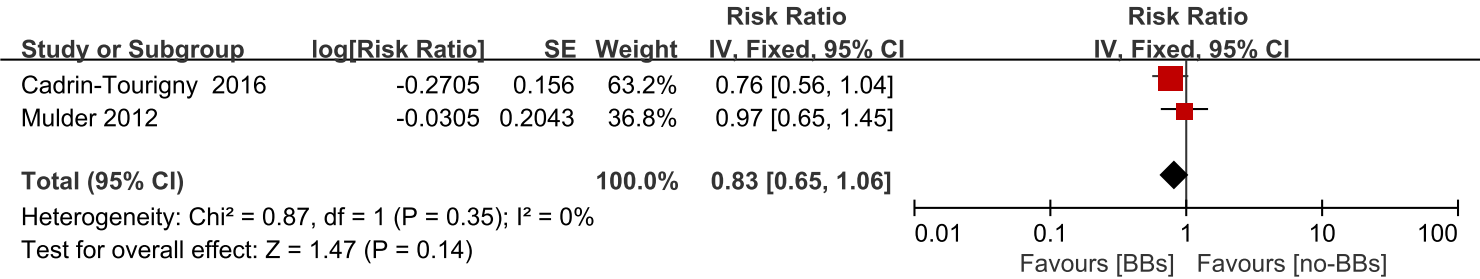

Supplement: Supplementary file 1 — Frost plot of the comparison of β-blockers treatment versus no β-blockers treatment in patients with chronic heart failure and atrial fibrillation, outcomes: cardiovascular mortality. BBs: β-blockers; CI: confidence intervals; SE: standard error. (PDF 212 kb) [file 12872_2019_1079_MOESM1_ESM.pdf]

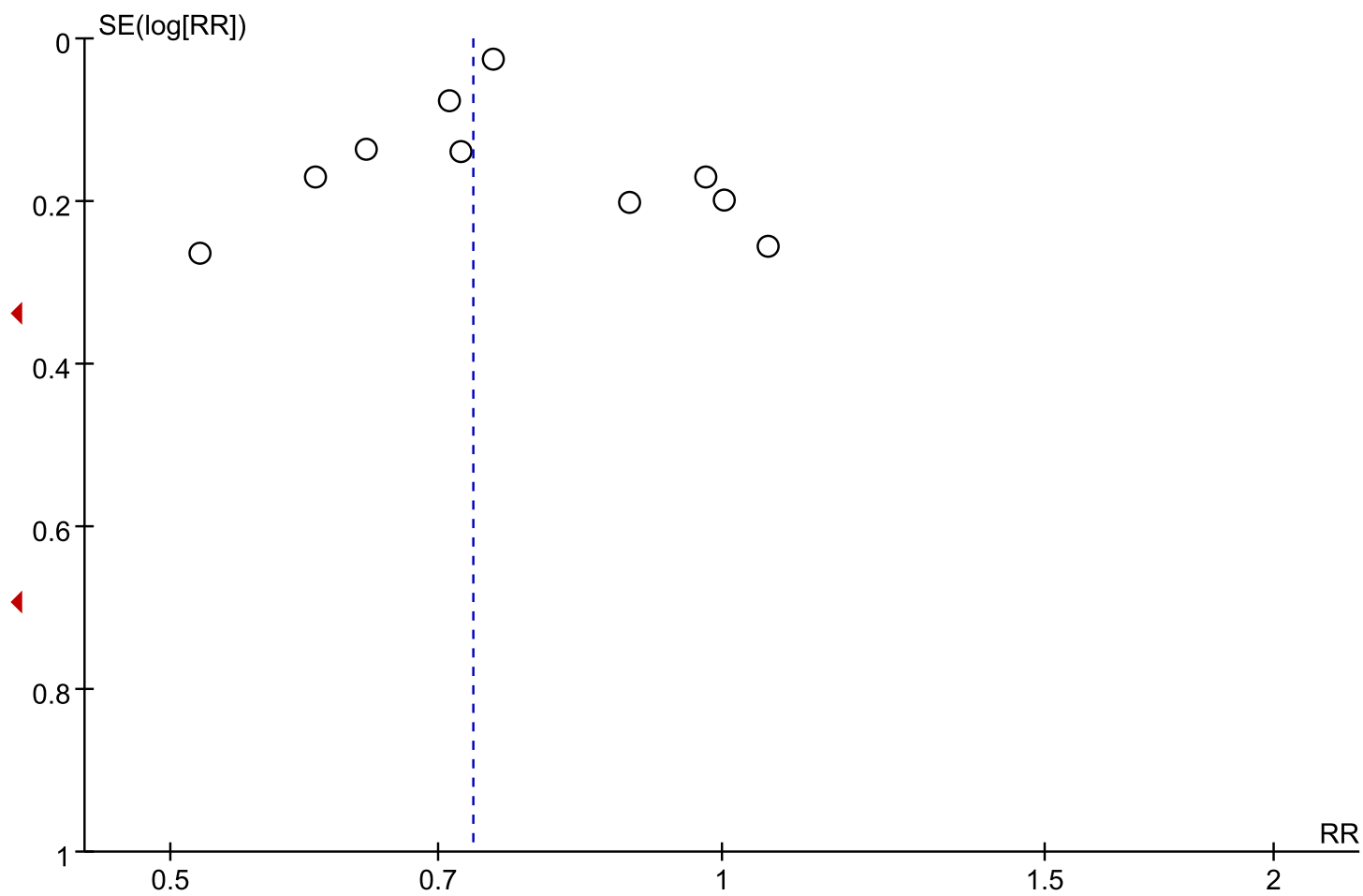

Supplement: Supplementary file 2 — Funnel plot of the comparison of β-blockers treatment versus no β-blockers treatment in patients with chronic heart failure and atrial fibrillation, outcomes: all-cause mortality. BBs: β-blockers; CI: confidence intervals; SE: standard error. (PDF 29 kb) [file 12872_2019_1079_MOESM2_ESM.pdf]

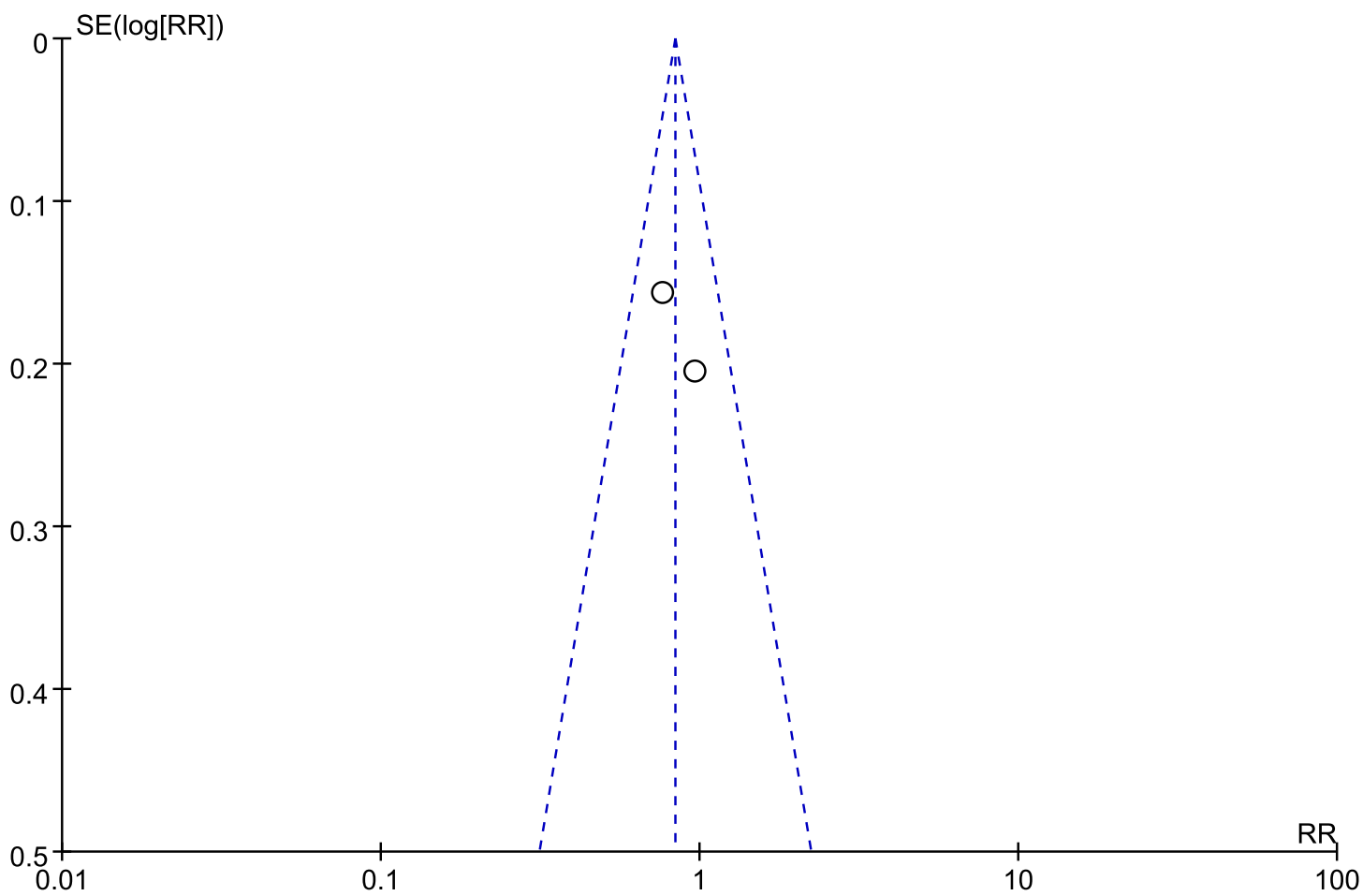

Supplement: Supplementary file 3 — Funnel plot of the comparison of β-blockers treatment versus no β-blockers treatment in patients with chronic heart failure and atrial fibrillation, outcomes: cardiovascular mortality. BBs: β-blockers; CI: confidence intervals; SE: standard error. (PDF 28 kb) [file 12872_2019_1079_MOESM3_ESM.pdf]

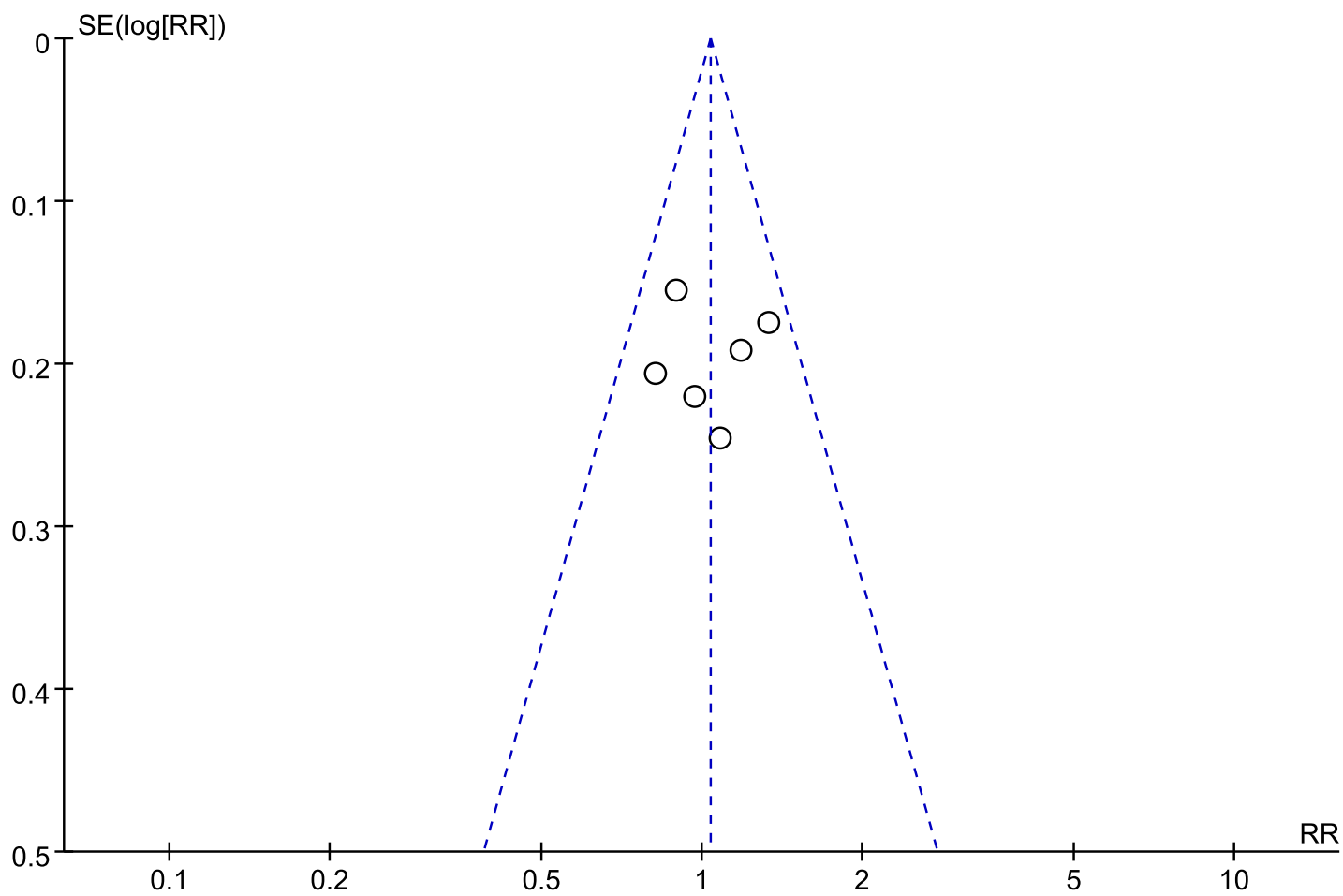

Supplement: Supplementary file 4 — Funnel plot of the comparison of β-blockers treatment versus no β-blockers treatment in patients with chronic heart failure and atrial fibrillation, outcomes: all-cause mortality. BBs: β-blockers; CI: confidence intervals; SE: standard error. (PDF 30 kb) [file 12872_2019_1079_MOESM4_ESM.pdf]

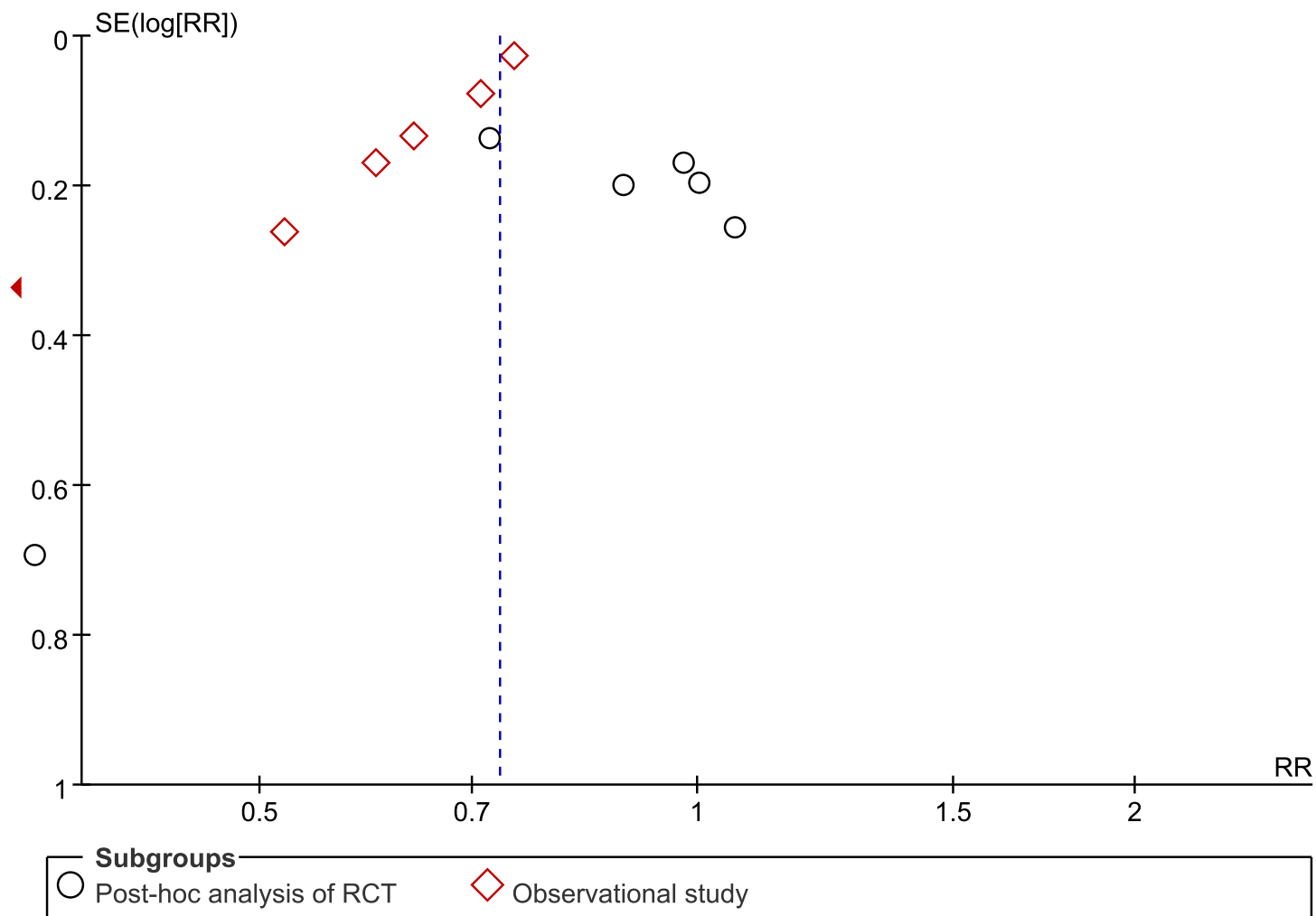

Supplement: Supplementary file 5 — Funnel plot the comparison of β-blockers treatment versus no β-blockers treatment in patients with chronic heart failure and atrial fibrillation. Stratified on study design, outcomes: all-cause mortality. BBs: β-blockers; CI: confidence intervals; SE: standard error. (PDF 67 kb) [file 12872_2019_1079_MOESM5_ESM.pdf]

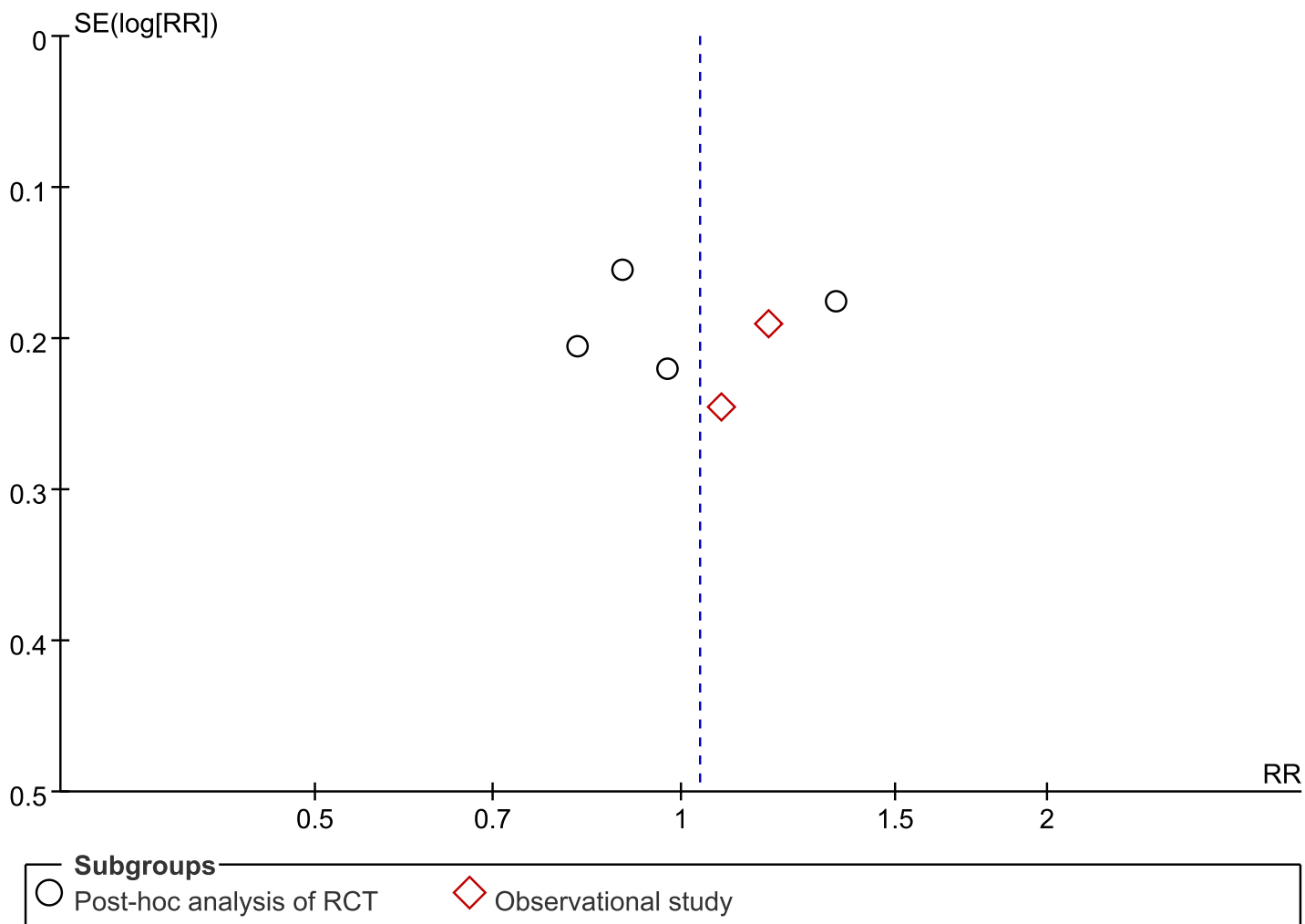

Supplement: Supplementary file 6 — Funnel plot of the comparison of β-blockers treatment versus no β-blockers treatment in patients with chronic heart failure and atrial fibrillation. Stratified on study design, outcomes: heart failure hospitalization. BBs: β-blockers; RCT: randomized control trial; CI: confidence intervals; SE: standard error. (PDF 67 kb) [file 12872_2019_1079_MOESM6_ESM.pdf]

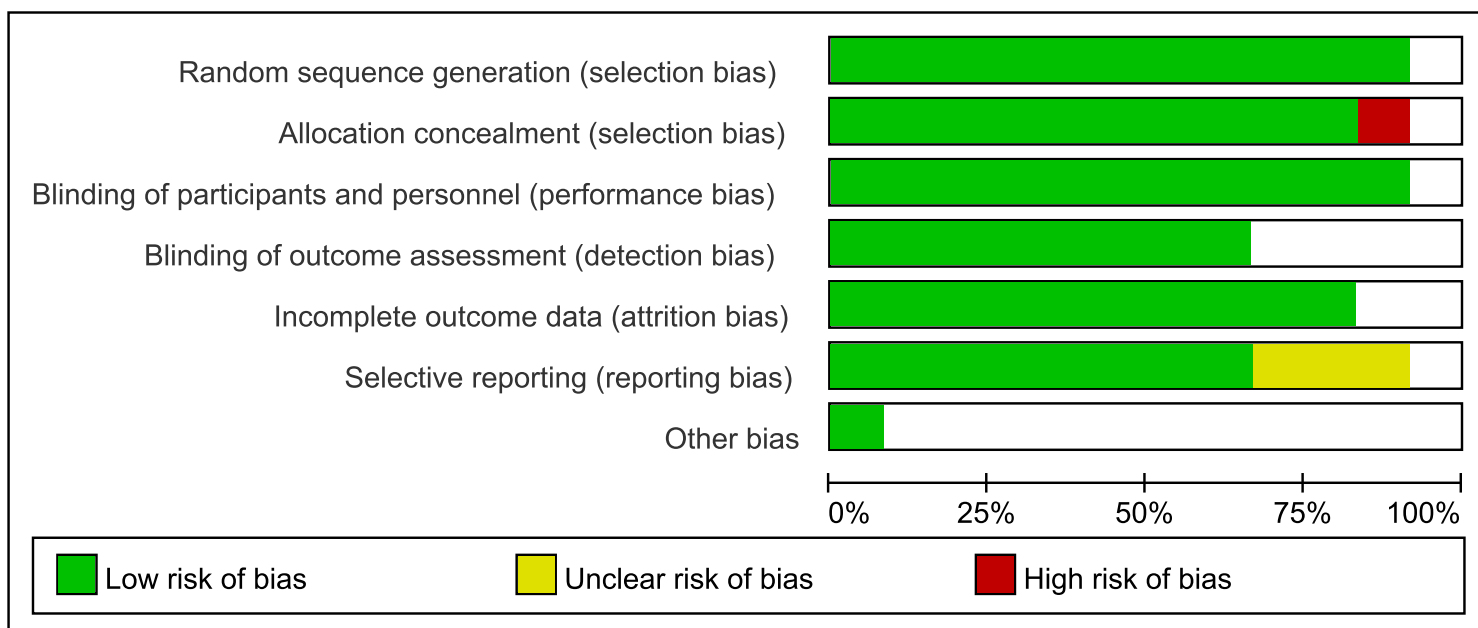

Supplement: Supplementary file 7 — Risk of bias graph. (PDF 238 kb) [file 12872_2019_1079_MOESM7_ESM.pdf]
