# Supplementary material for: β-blockers and risk of all-cause mortality in patients with chronic heart failure and atrial fibrillation—a meta-analysis
Source: BMC Cardiovasc Disord. 2019 Jun 3;19:135. doi: 10.1186/s12872-019-1079-2 (PMC6547467; doi:10.1186/s12872-019-1079-2)
Supplement: Supplementary file 8 — Search strategy for pubmed. (DOCX 14 kb) [file 12872_2019_1079_MOESM8_ESM.docx]

**S8 Search strategy for pubmed**

#1  Atrial fibrillation[MeSH Terms]

#2  Heart failure[MeSH Terms] OR heart failure, diastolic[MeSH Terms] OR heart failures, systolic[MeSH Terms]

#3 Bisoprolol[MeSH Terms] OR nebivolol[MeSH Terms] OR Metoprolol[MeSH Terms] OR Atenolol[MeSH Terms] OR adrenergic beta antagonists[MeSH Terms]

#4 #1 AND #2 AND #3

#5  Atrial fibrillation[Text Word]

#6 Heart failure[Text Word] OR Systolic heart failure[Text Word] OR Diastolic heart failure[Text Word] OR HFpEF[Text Word] OR HFrEF[Text Word] OR cardiac dysfunction[Text Word] OR heart dysfunction[Text Word] OR cardiac failure[Text word] OR heart weakness [Text Word]

#7 adrenergic beta antagonists[Text Word] OR Beta blockers[Text Word] OR Bisoprolol[Text Word] OR nebivolol[Text Word] OR carvedilol[Text Word] OR bucindolol[Text Word] OR Metoprolol[Text Word] OR Atenolol[Text Word] OR metoprolol CR/XL[Text Word]

#8 #5 AND #6 AND #7

#9 #4 OR #8

#10  Animals[MeSH Terms]

#11  Humans[MeSH Terms]

#12 #10 NOT #11

#13 #9 NOT #12
