# Supplementary material for: β-blockers and risk of all-cause mortality in patients with chronic heart failure and atrial fibrillation—a meta-analysis
Source: BMC Cardiovasc Disord. 2019 Jun 3;19:135. doi: 10.1186/s12872-019-1079-2 (PMC6547467; doi:10.1186/s12872-019-1079-2)
Supplement: Supplementary file 9 — Search strategy for embase. (DOCX 13 kb) [file 12872_2019_1079_MOESM9_ESM.docx]

**S9 Search strategy for embase**

#1 'atrial fibrillation':kw

#2 'beta blockers':kw OR 'adrenergic beta antagonists':kw

#3 'systolic heart failure':kw OR 'diastolic heart failure':kw OR 'heart failure':kw OR 'hfpef':kw OR 'hfref':kw OR 'cardiac dysfunction':kw OR 'heart dysfunction':kw OR ' cardiac failure ':kw OR 'heart weakness':kw

#4 'mortality':kw OR 'fatal outcome':kw OR 'death':kw

#5 #1 AND #2 AND #3 AND #4

#6 'atrial fibrillation'/exp

#7 'heart failure'/exp OR 'systolic heart failure'/exp OR 'diastolic heart failure'/exp

#8 'beta adrenergic receptor blocking agent'/exp

#9 'mortality'/exp OR 'fatality'/exp OR 'death'/exp

#10 #6 AND #7 AND #8 AND #9

#11 #5 OR #10

#12 (#5 OR #10) AND [humans]/lim
