# Supplementary material for: β-blockers and risk of all-cause mortality in patients with chronic heart failure and atrial fibrillation—a meta-analysis
Source: BMC Cardiovasc Disord. 2019 Jun 3;19:135. doi: 10.1186/s12872-019-1079-2 (PMC6547467; doi:10.1186/s12872-019-1079-2)
Supplement: Supplementary file 10 — Tests for Publication Bias. (DOCX 16 kb) [file 12872_2019_1079_MOESM10_ESM.docx]

**S10 Tests for Publication Bias**

Begg's Test

adj. Kendall's Score (P-Q) = -13

Std. Dev. of Score = 12.85

Number of Studies = 11

z = -1.01

Pr > |z| = 0.312

z = 0.93 (continuity corrected)

Pr > |z| = 0.350 (continuity corrected)

Egger's test

------------------------------------------------------------------------------

Std_Eff | Coef. Std. Err. t P>|t| [95% Conf. Interval]

-------------+----------------------------------------------------------------

slope | -.2725195 .0440179 -6.19 0.000 -.372095 -.1729441

bias | -.5196216 .5525631 -0.94 0.372 -1.769606 .7303628
